# Raman Spectroscopy Enables Confirmatory Diagnostics of Fusarium Wilt in Asymptomatic Banana

**DOI:** 10.3389/fpls.2022.922254

**Published:** 2022-06-15

**Authors:** Stephen Parlamas, Paul K. Goetze, Dillon Humpal, Dmitry Kurouski, Young-Ki Jo

**Affiliations:** ^1^Department of Biochemistry and Biophysics, Texas A&M University, College Station, TX, United States; ^2^Department of Plant Pathology and Microbiology, Texas A&M University, College Station, TX, United States; ^3^Institute for Advancing Health Through Agriculture, Texas A&M University, College Station, TX, United States

**Keywords:** Raman spectroscopy, Fusarium wilt, banana, diagnostics, *Fusarium oxysporum* f. sp. *cubense*

## Abstract

*Fusarium oxysporum* f. sp. *cubense* (FOC) causes Fusarium wilt, one of the most concerning diseases in banana (*Musa* spp.), compromising global banana production. There are limited curative management options after FOC infections, and early Fusarium wilt symptoms are similar with other abiotic stress factors such as drought. Therefore, finding a reliable and timely form of early detection and proper diagnostics is critical for disease management for FOC. In this study, Portable Raman spectroscopy (handheld Raman spectrometer equipped with 830 nm laser source) was applied for developing a confirmatory diagnostic tool for early infection of FOC on asymptomatic banana. Banana plantlets were inoculated with FOC; uninoculated plants exposed to a drier condition were also prepared compared to well-watered uninoculated control plants. Subsequent Raman readings from the plant leaves, without damaging or destroying them, were performed weekly. The conditions of biotic and abiotic stresses on banana were modeled to examine and identify specific Raman spectra suitable for diagnosing FOC infection. Our results showed that Raman spectroscopy could be used to make highly accurate diagnostics of FOC at the asymptomatic stage. Based on specific Raman spectra at vibrational bands 1,155, 1,184, and 1,525 cm^−1^, Raman spectroscopy demonstrated nearly 100% accuracy of FOC diagnosis at 40 days after inoculation, differentiating FOC-infected plants from uninoculated plants that were well-watered or exposed to water deficit condition. This study first reported that Raman spectroscopy can be used as a rapid and non-destructive tool for banana Fusarium wilt diagnostics.

## Introduction

*Fusarium oxysporum* is noted to be one of the most significant plant-pathogenic fungi, with various formae speciales able to infect over 100 different hosts including important crops (Dean et al., 2012). *Fusarium oxysporum* f. sp. *cubense* (FOC) is the main causal agent of Fusarium wilt or Panama disease in banana (*Musa* spp.; Ploetz, 2006) and considered as the most economically important fungal pathogen in current banana production. A new tropical race 4 of FOC (FOC TR4) has been found to cause high mortality of Cavendish cultivars, the primary commercial and most internationally traded type of banana. This fatal banana disease by FOC TR4 continues spreading in major banana-producing areas of the world (Ploetz, 2006; Garcia-Bastidas et al., 2019), predisposing banana industries to high risk of yield losses (Ploetz, 2006).

The initial symptoms of Fusarium wilt by FOC TR4 are similar to other types of stresses. The xylem of the roots takes a reddish-brown color at the initial site of infection. Vascular discoloration progresses through the rhizome and ultimately arrives in the pseudostem. As the pathogen grows in the plant vascular system, the flow of water and nutrients is restricted causing older leaves to begin yellowing, wilting, and splitting. Later, younger leaves yellow, and the whole plant completely wilts (Ploetz, 2006).

There are currently limited options for Fusarium wilt management in banana. Cavendish cultivars are generally susceptible to FOC TR4, while few cultivars with resistance are available. Fungicide application and soil fumigation delay the fungal progression in the short term but cannot hold its encroachment in the long term. Once infected, FOC cannot be eliminated from infected plants or soils (Ploetz, 2006). Chlamydospores or plant materials parasitized by mycelium of FOC can survive up to 30 years outside of living banana plants, making it difficult to eradicate the disease from infected fields (Stover, 1962). Thus far, the only reliable controls have been preventative measures, including quarantine, sterilization of equipment, and burning infected plants and fields (Ghag et al., 2015).

Given this lack of curative management solutions for Fusarium wilt, proper diagnosis in plants is even more imperative. Current diagnostic testing of FOC depends on the polymerase chain reaction (PCR) method. While the PCR method is reliable, it requires extraction and purification of high-quality DNA, which is often difficult to acquire and takes time because pathogen isolation from infected tissue is necessary. Delayed test results may lead to greater spread of plant diseases (Schaad et al., 2003). Further complicating matters, banana leaves do not exhibit Fusarium wilt symptoms until the plant has become irrevocably damaged (Lin et al., 2020). The current best diagnostics of FOC conducted with asymptomatic plants at the early infection stage requires taking samples from pseudostems; however, this sampling process is destructive, after which the plants may be destroyed.

Raman spectroscopy (RS) is a modern analytical technique that provides information about molecular vibrations and, consequently, the structure and composition of the analyzed samples (Virkler and Lednev, 2009; Yeturu et al., 2016; Kadam et al., 2017; Mandrile et al., 2019; Gupta et al., 2020). The Raman effect is based on a phenomenon of inelastic light scattering of photons by molecules that are being excited to higher vibrational or rotational states. (Long, 2002; Farber et al., 2019a; Payne and Kurouski, 2021). A growing body of evidence shows that RS can detect mutations in a plant’s DNA or RNA down to a single molecule (Kadam et al., 2014, 2017). Previous studies have demonstrated that RS could detect plant biochemistry resulting from biotic and abiotic stresses in plants ranging from roses to tomatoes to wheat to oranges (Farber et al., 2019a; Payne and Kurouski, 2021). Raman scattering changes in plants intrigued by pathogen infection or environmental stresses could be detected and differentiated by RS (Farber and Kurouski, 2018), allowing for the confirmatory diagnostics of plant diseases (Farber et al., 2019a, 2020; Sanchez et al., 2020a,b,c).

Given the plausibility of RS application to plant disease detection, the objective of this study is to develop a non-destructive RS method for diagnosing early FOC infection in asymptomatic banana plants.

## Materials and Methods

### FOC Inoculum Preparation

FOC TR4 culture (ATCC^®^ 96289™) was obtained from the American Type Culture Collection, Manassas, Virginia, United States. The origin is an isolate from Cavendish banana in southeastern Queensland, Australia (Moore et al., 1991). Mung bean broth was used to produce conidia for inoculation (Garcia-Bastidas et al., 2019). Mung beans (20 g) were boiled in a 1 L flask containing 500 ml water for 35 min and cooled down. This broth was corked with a cotton and cheesecloth plug, autoclaved for 1 h, and allowed to cool to room temperature. At this point, five agar plugs approximately 2.5 cm by 2.5 cm from actively growing FOC mycelium on potato dextrose agar medium were added to the flask, which then was placed and incubated in an orbital shaker (Model 5000IR, VWR, Radnor, PA) at 25°C and a 150-rpm rotating speed. After 6 days, the liquid culture was strained out using miracloth (Electron Microscopy Sciences, Hatfield, Pennsylvania, United States), and conidia were collected. Harvested conidia were diluted to 10^6^ conidia ml^−1^.

### Treatments and Greenhouse Conditions

Dwarf Cavendish banana offshoots (3-leaf stage) were obtained from Hello Organics, Apopka, Florida, United States. Plants were transferred to one-gallon pots with potting soil (Jolly Gardener Products, Proline C/25 Potting Mix, Atlanta, Georgia, United States) and kept in a greenhouse conditioning as 27–30°C, 25–50% humidity, and a 12-h natural light cycle. Watering using reverse osmosis treated water (RO water) was provided twice a day plus a 4-h misting to increase humidity in the afternoon. Liquid fertilizer (Miracle Grow All Purpose Plant Food, Marysville, Ohio, United States) was added at the first day of each month.

Nine banana plants were divided into three groups of three plants and randomly assigned with three treatments: (i) uninoculated and well-watered with daily irrigation (Control group), (ii) FOC-inoculated and well-watered with daily irrigation (FOC group), and (iii) uninoculated and water-deficient with every other day irrigation (Drought group). Average of volumetric water content in the soil was 42.8% before watering and 50% after watering under daily irrigation regime; and 17.6% before watering and 35% after watering under every other day regime. The FOC group was inoculated with 200 ml of FOC conidial solution (10^6^ conidia ml^−1^) per pot (Garcia-Bastidas et al., 2019). All environmental conditions remained the same for the control and FOC groups. The drought group had non-inoculated plants that were exposed to the water deficit condition. After exposure to the same daily irrigation regime, the plants of the drought group were moved away from the misting area and given water every other day starting at 1 month after inoculation (DAI), the same day Raman spectroscopy measurement on samples began to be taken.

Upon the conclusion of the experiment after about 3 months, banana plants were harvested to confirm infections in plants. Plant pseudostems were split to determine symptom development in the early infection stage. Photographs of the pseudostem cross-sections were taken, and symptomatic areas showing discoloration were measured using ImageJ.JS software (Ouyang et al., 2019) to give a quantitative value to the disease.

The experiment was completely randomized design with three replicates (the first trial) and five replicates (the second trial).

### Raman Spectroscopy

RS was conducted weekly for examining plants starting 33 DAI and continued until 75 DAI. Raman spectra were taken from plants (top three fully expanded leaves in the areas between the leaf veins) with a handheld Resolve Agilent spectrometer equipped with 830 nm laser source. Over the course of the whole experiment, spectra were taken in the same location and at approximately the same time on the day. Locations for spectral acquisition were randomly taken across plant leaves; 2–3 spectra were taken per one plant leaf. In total, 50 spectra were collected per sample (Control, FOC and Drought groups) per day. The following experimental parameters were used for all collected spectra: 1 s acquisition time, 495 mW power, and baseline spectral subtraction by device software (Agilent, Santa Clara, CA, United States). Fifty spectra were collected from each group of plants at the given timepoint; no distinctions were made for individual plants within each group.

### Multivariate Data Analysis

PLS_Toolbox software (Eigenvector Research Inc., Manson, Washington, United States) was used for the statistical analysis of collected Raman spectra. First, derivative was taken from raw Raman spectra with a filter width of 45 and polynomial order 2, and spectra were median centered. Partial least squares discriminant analysis (PLS-DA) was performed to determine the number of significant components and identify spectral regions that best explain the separation between the three groups of banana plants. To give each of the spectral regions with equal importance, all spectra were scaled to unit variance. Raw spectra, containing wavenumbers 350–2000 cm^−1^, were retained in the model that resulted from this iteration of PLS-DA. In both trials, bananas were grown and inoculated identically, and Raman spectra were collected weekly starting at 33 DAI at the same time of day to eliminate controllable variation between classes and trials. However, some variation in banana was left unaltered in the statistical analysis such as randomly chosen locations of laser scans on the leaves to demonstrate the repeatability of the results. In parallel, Kruskal–Wallis one-way analysis of variance was used to determine the significance of changes in intensities of the observed vibrational bands. Kruskal–Wallis one-way analysis tests whether the median in a set of samples is significantly different from other classes. The null hypothesis of this test was that there was no significant difference at the band of interests. The significant level (α) was 0.05. The Kruskal–Wallis also reported a 95% confidence interval for the true value of median for each compared group.

## Results

### Disease Progression

**Figure 1:**
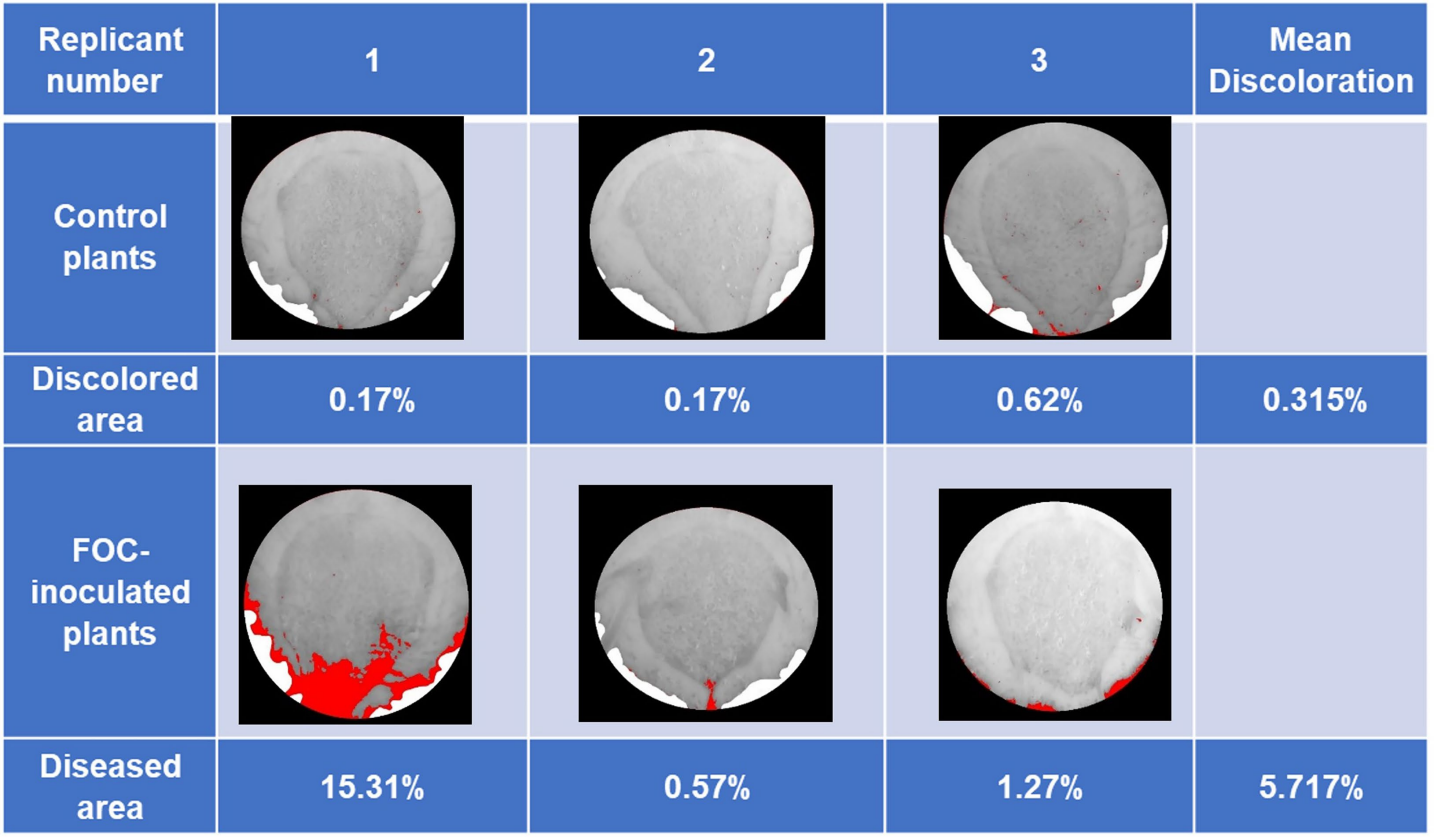
Banana Fusarium symptom development in pseudostem cross-sections during the first trial at 89 days after inoculation (DAI). Pure white areas are cover for areas in the image that are not part of the pseudostem. Percentage of diseased areas (denoted by large continuous red areas in the images) was measured in FOC-inoculated plants using ImageJ.JS software, showing discoloration caused by disease infection. Red areas were shown in the Control group due to shadow, soil particulate, and other factors darkening parts of the images because ImageJ.JS software only distinguishes color by pixel. The Drought group plants were not harvested for pseudostem examination in this trial.

Early disease symptoms in leaves such as yellowing and drooping started to exhibit themselves at 75 DAI of the first trial. At the end of experiment (89 DAI), FOC-inoculated plants had an average discolored area of 5.717% in pseudostem cross-sections (dark black and brown necrotic tissues compared to healthy white and light-yellow tissue; Figure 1). Minor discolored areas shown in pseudostems images of the Control group were also detected due to shadow, soil particulate, and other slight darkening, as the ImageJ.JS software only distinguished color by pixel. Drought-stressed plants increased yellowing of upper leaves compared to Control plants at 75 DAI but similar to FOC-inoculated plants. Raman samples were taken from the Drought group throughout the experiment; however, this group was not harvested for pseudostem examination.

**Figure 2:**
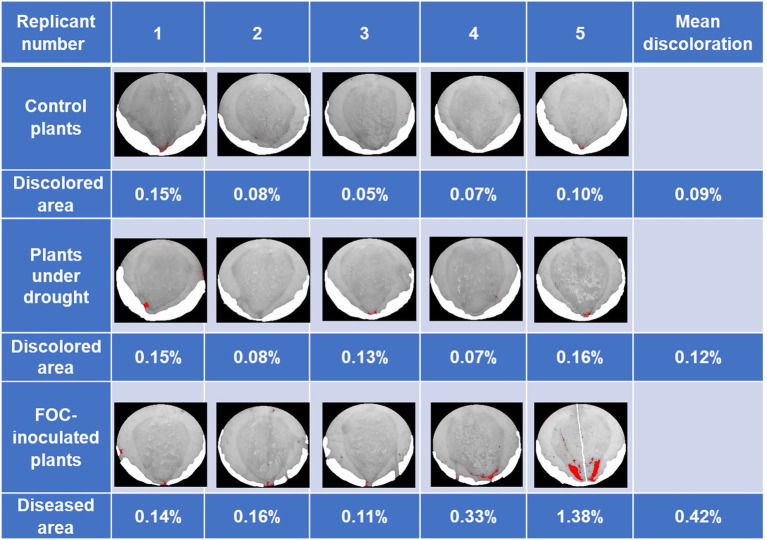
Banana Fusarium symptom development in pseudostem cross-sections during the second trial at 79 days after inoculation (DAI). Pure white areas are cover for areas in the image that art not part of the pseudostem. Percentage of diseased areas (denoted by large continuous red areas in the images) was measured in FOC-inoculated plants using ImageJ.JS software, showing discoloration caused by disease infection. Red areas were shown in the Control and Drought group due to shadow, soil particulate, and other factors darkening parts of the images because ImageJ.JS software only distinguishes color by pixel.

The second trial showed similar results. Early symptoms of yellowing in leaves of plants in both Drought and FOC groups began to be exhibited at 72 DAI; plants were harvested at this time before clear symptoms appeared. FOC-inoculated plants showed average 0.42% of discoloration in pseudostem cross-section areas (Figure 2). As with the first trial, Control and Drought group plant images showed minor darker spots that were not caused by FOC infection.

### Raman-Based Diagnostics of Fusarium Wilt

Raman spectra determined three different treatments of Control, FOC, and Drought groups at the accuracy of 76, 88, and 85%, respectively, as early as 40 DAI, when disease symptoms on leaves were not developed (Table 1). The detection accuracy continuously increased as the disease progressed on later DAI and reached >90% on 61–75 DAI. From 40 DAI, FOC-inoculated plants could be differentiated from Drought group at 99–100% accuracy.

**Table 1:** Accuracy of binary models for determining three groups of banana plants.

**Group**	**Treatment**	**Days after FOC inoculation**					
		**40**	**47**	**54**	**61**	**68**	**75**
Control group	Uninoculated and well-watered	76%	82%	76%	92%	100%	89%
FOC group	FOC-inoculated and well-watered	88%	94%	81%	94%	90%	94%
Drought group	Uninoculated and water-deficient	85%	76%	81%	100%	100%	94%
Comparison between FOC group and Drought group		100%	99%	100%	100%	100%	99%

**Figure 3:**
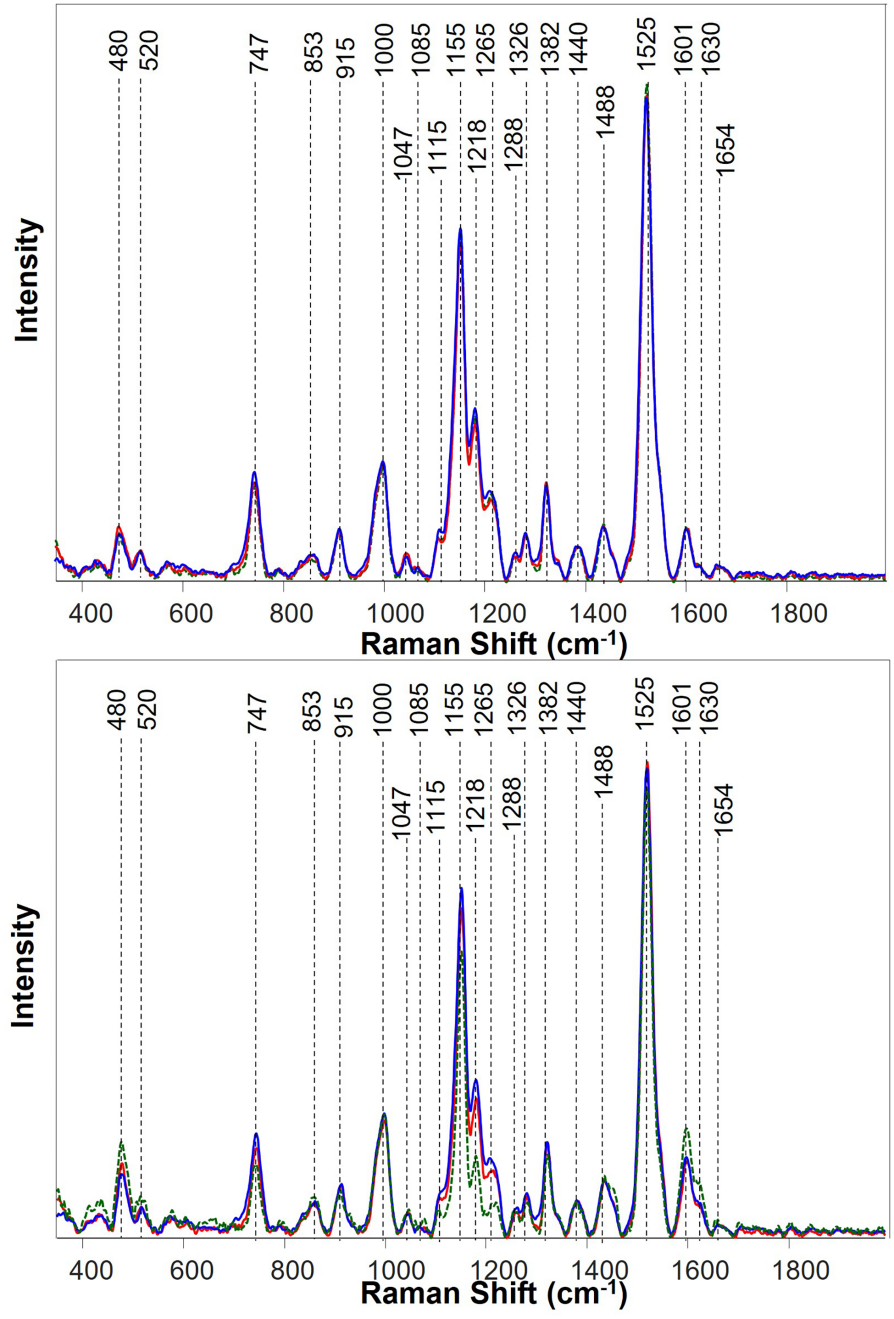
Averaged Raman spectra of banana leaves at the early infection at 47 days after inoculation (DAI; top) and asymptomatic plants at 75 DAI (bottom). Three treatment groups were color-coded as uninoculated and well-watered control plants (red), FOC-inoculated and well-watered plants (blue), and uninoculated and water-deficient plants (green).

Spectra collected from banana leaves exhibited vibrational bands that could be assigned to four classes of chemical compounds: (i) carbohydrates, including cellulose (480, 520, 853, 915, and 1,047 cm^−1^) and pectin (747 cm^−1^), (ii) carotenoids (1,000, 1,085, 1,115, 1,155, 1,184, 1,218, 1,525, and 1,545 cm^−1^), and (iii) Phenylpropanoids (1,601 and 1,630 cm^−1^), and (iv) proteins (1,654 cm^−1^; Table 2 and Figure 3). Other vibrational bands observed in this study were assigned to aliphatic (C–H and CH_2_) vibrations (1,288, 1,326, 1,382, 1,440, and 1,488 cm^−1^). However, these chemical moieties are present in nearly all classes of biological molecules and therefore could not be assigned to a certain class of chemical compounds.

**Table 2:** Vibrational bands and their assignments for Raman spectra collected from banana plants.

Band (Raman Shift, cm^−1^)	Vibrational mode	Assignment
480–520	CCO and CCC deformations; related to glycosidic ring skeletal deformations δ(C−C−C)+(C−O) scissoring of C−C−C and out-of-plane bending of C−O	Cellulose (Edwards et al., 1997)
747	ν(C–O–H) of COOH	Pectin (Synytsya et al., 2003)
853–915	ν(C–O–C) in plane, symmetric	Cellulose (Edwards et al., 1997)
1,000	ν(C–CH_3_ stretching) and phenylalanine	Carotenoids (Tschirner et al., 2009; Kurouski et al., 2015)
1,047	ν(C–O)+ν(C–C)+δ(C–O–H)	Cellulose (Almeida et al., 2010)
1,085–1,218	ν(C–CH_3_ stretching) and phenylalanine	Carotenoids (Tschirner et al., 2009; Kurouski et al., 2015)
1,265	δ(C–C–H)	Aliphatic (Yu et al., 2007)
1,288	δ(C–C–H)	Aliphatic (Yu et al., 2007)
1,326	δCH_2_ bending vibration	cellulose, lignin (Edwards et al., 1997)
1,382	δCH_2_ bending vibration	Aliphatic (Yu et al., 2007)
1,440	δ(CH_2_)+δ(CH_3_)	Aliphatic (Yu et al., 2007)
1,488	δ(CH_2_)+δ(CH_3_)	Aliphatic (Yu et al., 2007)
1,527–1,545	–C=C– (in plane)	Carotenoids (Adar, 2017; Devitt et al., 2018)
1,601–1,604	ν(C–C) aromatic ring+(CH)	Phenylpropanoids (Agarwal, 2006; Kang et al., 2016)
1,654	C=O stretching, amide I	Proteins (Devitt et al., 2018)

*δ*: symmetric bending vibration; *ν*: stretching vibration.

Raman spectra collected from pre-symptomatic plants at 47 DAI were mostly similar among different treatment groups (Figure 3). Nevertheless, a small increase in the Raman spectral intensity of carotenoids was detected in plants inoculated with FOC or under drought stress, compared with the uninoculated well-watered control plants.

At 75 DAI, plants exhibited drastically different spectra among different groups of banana plants. In the drought group, comparing with the control group, a drastic decrease in the Raman intensity of carotenoids and pectin had taken place, while the Raman intensity for cellulose and phenylpropanoids increased. In case of the FOC group, compared with the control group and drought group, the Raman intensity of carotenoids and pectin increased, but that of cellulose decreased.

Kruskal–Wallis analysis of the vibrational bands at 1155, 1184, and 1,525 cm^−1^, which can be assigned to carotenoids, determined statistically significant changes in their Raman intensities among the three different groups of plants. Specifically, our results showed RS collected from Drought-stressed plants exhibited significantly lower intensities of carotenoid bands compared to the intensity of these bands in the spectra of healthy (Control) plants. The opposite change in the intensity of these bands was found for FOC plants. Specifically, the intensity of carotenoid bands was found to be greater in the spectra collected from FOC plants compared to the intensity of 1,155, 1,184 and 1,525 cm^−1^ bands in the spectra of healthy (Control) plants. Thus, Kruskal–Wallis analysis demonstrate that carotenoids bands can be used as a marker for diagnostics of Drought and FOC stresses in banana.

## Discussion

The lack of early symptom development of Fusarium wilt likely results in unrecognized infection and brewing disease progress under the ground. During this critical window of time, between initial infection and obvious symptom development, implementation of disease management practices relies on prompt, fast, and practical diagnostics. Improper or delayed diagnostics give FOC an opportunity to decimate plants and spread to others. A conventional method currently used for FOC testing is to sacrifice plants to examine pseudostems for the presence of rotting symptoms, but such a destructive method is not ideal for preserving plants.

The current study shows that RS can be used to detect early infection stage or low disease severity of Fusarium wilt in banana. This approach is label-free because labeling target compounds is not necessary, non-invasive because the introduction of instruments into plant tissue is not required, and non-destructive because tested leaves or plants are not damaged. This novel detection and identification method of FOC in banana provides a major step toward practical diagnostics of Fusarium wilt in an asymptomatic stage. The early diagnosis using RS may be able to provide better options to manage Fusarium wilt in banana fields. Previous research has evaluated the applicability of RS for detecting banana Fusarium wilt (Lin et al., 2020). Although proving the ability of RS to detect signatures unique to Fusarium wilt at an early stage in disease progress, the method used in the earlier study still relied on pseudostem tissue for early detection.

**Figure 4:**
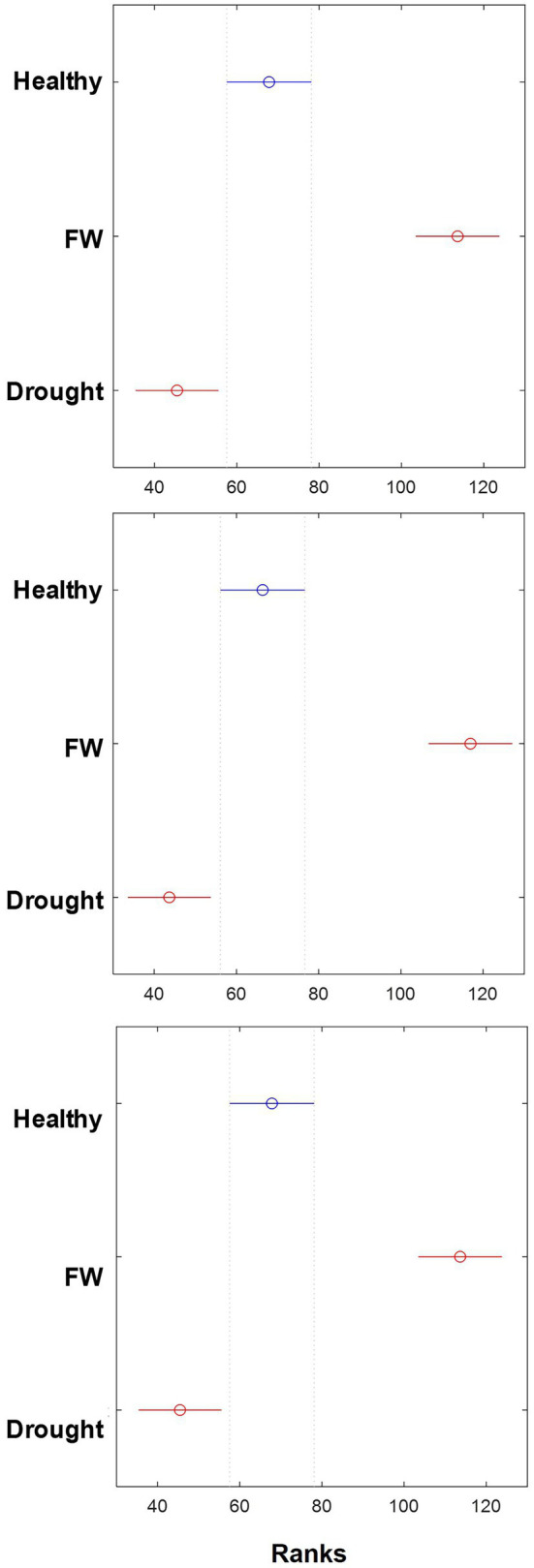
Kruskal–Wallis plots of Raman intensity of 1,155 cm^−1^ (top), 1,184 cm^−1^ (center), and 1,525 cm^−1^ (bottom) bands in the spectra collected from three treatment groups: uninoculated and well-watered control plants (Control group), FOC-inoculated and well-watered plants (FOC group), and uninoculated and water-deficient plants (Drought group).

Different patterns of Raman spectra associated with banana plants under biotic and abiotic stresses suggested that banana enhanced carotenoid production as a response to FOC infection and drought stress. Therefore, the differential intensity of carotenoids vibrations can be used as a marker band for confirmatory differentiation of FOC-infected plants from uninfected well-watered plants and from uninfected plants under drought stress. Kruskal–Wallis analysis of the Raman intensity of three carotenoid bands confirmed this hypothesis (Figure 4). These Raman spectral changes indicate that drastically different biochemical reactions of banana plants are occurring while responding to biotic and abiotic stresses. Carotenoids are known to be transformed into signaling molecules such as abscisic acid that are directly involved in the plant’s response to the biotic and abiotic stress (Parry and Horgan, 1991). Our results also suggest that drought stress causes an increase in the concentration of either low molecular weight phenylpropanoids such as cinnamic acid or aromatic polymers such as lignin. Advance chromatographic and mass spectroscopic analyses are required to determine changes associated with the biotic and abiotic stress on the molecular level. Such analyses are beyond the scope of the current study.

We hypothesized that these spectroscopic changes can be used for confirmatory detection and identification of biotic and abiotic stresses in banana. To test this hypothesis, we utilize PLS-DA to determine the accuracy of diagnostics of both FOC and drought condition relative to healthy control plants. We also question the accuracy of specificity of RS in differentiating FOC diagnostics from drought stresses since early symptoms on leaves are similar. Our results demonstrate that FOC and drought can be predicted differently as early as 40 DAI with 88 and 85% accuracy, respectively (Table 1). As the disease progressed after 61 DAI, the accuracy of identification of FOC and drought increased to >90%. We also found that in the early period when the watering schedule changed to every other day from daily irrigation, the accuracy of identification of drought plants was ~80%, which suggests that plants already experienced drought stress, although apparent drought symptoms on leaves such as drooping, or wilting, were not observed. However, 61 DAI and, thereafter, the accuracy of drought stress identification reached up to 100%.

The most significant outcome of this research is that these biotic and abiotic stresses can be diagnosed in the early infection stage (40-47 DAI) and the infection-progressing but asymptomatic plants (68–75 DAI) with high accuracy. RS enables nearly 100% accurate differentiation between FOC infection and drought stress in asymptomatic plants. These results demonstrate that RS can be used for highly accurate detection and identification of both FOC and drought stress in asymptomatic and early symptomatic banana plants while being non-destructive by taking readings from leaves and cost-effective since sample preparation and lab facility are not required. Although not a solution to ending FOC spread or curing infected plants, early diagnosis using RS can limit losses by timely implementing preventative quarantine, sterilization methods, and eradication of infected plants in more precise ways.

Our findings of characteristic Raman profiles shed light on the biochemical origin of these changes in plants. Specifically, the data indicate that drought causes a substantial decrease in the concentration of carotenoids, whereas FOC is on the opposite associated with an increase in the concentration of carotenoids. These spectroscopic changes can facilitate elucidation of molecular mechanisms of plant responses to such biotic and abiotic stresses. Considering high sensitivity of RS for diagnostics of biotic stresses on plants (Altangerel et al., 2017; Egging et al., 2018; Farber and Kurouski, 2018; Farber et al., 2019a,b; Sanchez et al., 2019a,b, 2020a), RS approach has far-reaching implications in various disciplines ranging from plant molecular biology to plant pathogen detection in agriculture and breeding.

## Data Availability Statement

The raw data supporting the conclusions of this article will be made available by the authors, without undue reservation.

## Author Contributions

SP, PG, DH, Y-KJ, and DK conceived and designed the study. PG and Y-KJ focused on the biological aspects including plant care, FOC culture maintenance, and inoculation of the plants as well as maintenance of the workspace. SP and DH took Raman spectroscopy samples and cared for the RS equipment. SP, DH, and DK conducted statistical analysis. SP and PG wrote the draft manuscript. All authors contributed to the article and approved the submitted version.

## Funding

This work was supported by the Texas A&M AgriLife Research and Governor’s University Research Initiative (GURI) grant program of Texas A&M University, GURI Grant Agreement (No. 12-2016, M1700437). DK acknowledges the Institute for Advancing Health Through Agriculture for providing financial support.

## Conflict of Interest

The authors declare that the research was conducted in the absence of any commercial or financial relationships that could be construed as a potential conflict of interest.

## Publisher’s Note

All claims expressed in this article are solely those of the authors and do not necessarily represent those of their affiliated organizations, or those of the publisher, the editors and the reviewers. Any product that may be evaluated in this article, or claim that may be made by its manufacturer, is not guaranteed or endorsed by the publisher.

## References

[ref1] Adar F. (2017). Carotenoids-their resonance Raman spectra and how they can be helpful in characterizing a number of biological systems. Spectroscopy 32, 12–20.

[ref2] Agarwal U. P. (2006). Raman imaging to investigate ultrastructure and composition of plant cell walls: distribution of lignin and cellulose in black spruce wood (*Picea Mariana*). Planta 224, 1141–1153. doi: 10.1007/s00425-006-0295-z, PMID: 16761135

[ref3] Almeida M. R. Alves R. S. Nascimbem L. B. Stephani R. Poppi R. J. de Oliveira L. F. (2010). Determination of amylose content in starch using Raman spectroscopy and multivariate calibration analysis. Anal. Bioanal. Chem. 397, 2693–2701. doi: 10.1007/s00216-010-3566-2, PMID: 20213166

[ref4] Altangerel N. Ariunbold G. O. Gorman C. Alkahtani M. H. Borrego E. J. Bohlmeyer D.. (2017). In vivo diagnostics of early abiotic plant stress response via Raman spectroscopy. Proc. Natl. Acad. Sci. U. S. A. 114, 3393–3396. doi: 10.1073/pnas.1701328114, PMID: 28289201 PMC5380084

[ref5] Dean R. van Kan J. A. L. Pretorius Z. A. Hammond-Kosack K. E. di Pietro A. Spanu P. D.. (2012). The top 10 fungal pathogens in molecular plant pathology. Mol. Plant Pathol. 13, 414–430. doi: 10.1111/J.1364-3703.2011.00783.X, PMID: 22471698 PMC6638784

[ref6] Devitt G. Howard K. Mudher A. Mahajan S. (2018). Raman spectroscopy: an emerging tool in neurodegenerative disease research and diagnosis. ACS Chem. Neurosci. 9, 404–420. doi: 10.1021/acschemneuro.7b00413 29308873

[ref7] Edwards H. G. Farwell D. W. Webster D. (1997). Ft Raman microscopy of untreated natural plant Fibres. Spectrochim. Acta A Mol. Biomol. Spectrosc. 53A, 2383–2392. doi: 10.1016/S1386-1425(97)00178-9, PMID: 9477578

[ref8] Egging V. Nguyen J. Kurouski D. (2018). Detection and identification of fungal infections in intact wheat and Sorghum grain using a hand-held Raman spectrometer. Anal. Chem. 90, 8616–8621. doi: 10.1021/acs.analchem.8b01863, PMID: 29898358

[ref9] Farber C. Bryan R. Paetzold L. Rush C. Kurouski D. (2020). Non-invasive characterization of single-, double- and triple-viral diseases of wheat with a hand-held Raman spectrometer. Front. Plant Sci. 11:1300. doi: 10.3389/fpls.2020.01300, PMID: 33013951 PMC7495046

[ref10] Farber C. Kurouski D. (2018). Detection and identification of plant pathogens on maize kernels with a hand-held Raman spectrometer. Anal. Chem. 90, 3009–3012. doi: 10.1021/acs.analchem.8b00222, PMID: 29461798

[ref11] Farber C. Mahnke M. Sanchez L. Kurouski D. (2019a). Advanced spectroscopic techniques for plant disease diagnostics. A Review. Trends Analyt. Chem. 118, 43–49. doi: 10.1016/j.trac.2019.05.022

[ref12] Farber C. Shires M. Ong K. Byrne D. Kurouski D. (2019b). Raman spectroscopy as an early detection tool for rose rosette infection. Planta 250, 1247–1254. doi: 10.1007/s00425-019-03216-0, PMID: 31222494

[ref13] Garcia-Bastidas F. A. van der Veen A. J. T. Nakasato-Tagami G. Meijer H. J. G. Arango-Isaza R. E. Kema G. H. J. (2019). An improved Phenotyping protocol for Panama disease in Banana. Front. Plant Sci. 10:1006. doi: 10.3389/fpls.2019.01006, PMID: 31447871 PMC6691145

[ref14] Ghag S. B. Shekhawat U. K. S. Ganapathi T. R. (2015). Fusarium wilt of Banana: biology, epidemiology and management. Int. J. Pest Manage. 61, 250–263. doi: 10.1080/09670874.2015.1043972

[ref15] Gupta S. Huang C. H. Singh G. P. Park B. S. Chua N.-H. Ram R. J. (2020). Portable Raman leaf-clip sensor for rapid detection of plant stress. Sci. Rep. 10:20206. doi: 10.1038/s41598-020-76485-5, PMID: 33214575 PMC7677326

[ref16] Kadam U. S. Chavhan R. L. Schulz B. Irudayaraj J. (2017). Single molecule Raman spectroscopic assay to detect Transgene from GM plants. Anal. Biochem. 532, 60–63. doi: 10.1016/j.ab.2017.06.002, PMID: 28602750

[ref17] Kadam U. S. Schulz B. Irudayaraj J. (2014). Detection and quantification of alternative splice sites in Arabidopsis genes AtDCL2 and AtPTB2 with highly sensitive surface Enahnced Raman spectroscopy (SERS) and gold Nanoprobes. FEBS Lett. 588, 1637–1643. doi: 10.1016/j.febslet.2014.02.061, PMID: 24631541

[ref18] Kadam U. S. Schulz B. Irudayaraj J. M. K. (2017). Multiplex single-cell quantification of rare RNA transcripts from protoplasts in a model plant system. Plant J. 90, 1187–1195. doi: 10.1111/tpj.13537, PMID: 28301688

[ref19] Kang L. Wang K. Li X. Zou B. (2016). High pressure structural investigation of benzoic acid: Raman spectroscopy and X-ray diffraction. J. Phys. Chem. C 120, 14758–14766. doi: 10.1021/acs.jpcc.6b05001

[ref20] Kurouski D. Van Duyne R. P. Lednev I. K. (2015). Exploring the structure and formation mechanism of amyloid fibrils by Raman spectroscopy: A review. Analyst 140, 4967–4980. doi: 10.1039/c5an00342c, PMID: 26042229

[ref21] Lin Y.-J. Lin H. -K. Lin Y. -H. (2020). Construction of Raman spectroscopic fingerprints for the detection of Fusarium wilt of Banana in Taiwan. PLoS One 15, 1–14. doi: 10.1371/journal.pone.0230330, PMID: 32176731 PMC7075571

[ref22] Long D. A. (2002). The Raman Effect: A Unified Treatment of the Theory of Raman Scattering by Molecules. England: West Sussex.

[ref23] Mandrile L. Rotunno S. Miozzi L. Vaira A. M. Giovannozzi A. M. Rossi A. M.. (2019). Nondestructive Raman spectroscopy as a tool for early detection and discrimination of the infection of tomato plants by two economically important viruses. Anal. Chem. 91, 9025–9031. doi: 10.1021/acs.analchem.9b01323, PMID: 31265250

[ref24] Moore N. Y. Hargreaves P. A. Pegg K. G. Irwin J. A. G. (1991). Characterisation of strains of *Fusarium oxysporum* F. Sp. *Cubense* by production of volatiles. Aust. J. Bot. 39, 161–166. doi: 10.1071/BT9910161

[ref25] Ouyang W. Mueller F. Hjelmare M. Lundberg E. Zimmer C. (2019). Imjoy: an open-source computational platform for the deep learning era. Nat. Methods 16, 1199–1200. doi: 10.1038/s41592-019-0627-0, PMID: 31780825

[ref26] Parry A. D. Horgan R. (1991). Carotenoids and Abscisic acid (aba) biosynthesis in higher plants. Physio. Plant. 82, 320–326. doi: 10.1111/j.1399-3054.1991.tb00100.x

[ref27] Payne W. Z. Kurouski D. (2021). Raman-based diagnostics of biotic and abiotic stresses in plants. A Review. Front Plant Sci. 11:616672. doi: 10.3389/fpls.2020.616672, PMID: 33552109 PMC7854695

[ref28] Ploetz R. C. (2006). Fusarium wilt of Banana is caused by several pathogens referred to as *Fusarium oxysporum* F. Sp. Cubense. Phytopathology 96, 653–656. doi: 10.1094/PHYTO-96-0653, PMID: 18943184

[ref29] Sanchez L. Ermolenkov A. Biswas S. Septiningshih E. M. Kurouski D. (2020a). Raman spectroscopy enables non-invasive and confirmatory diagnostics of salinity stresses, nitrogen, phosphorus, and potassium deficiencies in Rice. Front. Plant Sci. 11:573321. doi: 10.3389/fpls.2020.573321, PMID: 33193509 PMC7642205

[ref30] Sanchez L. Ermolenkov A. Tang X. T. Tamborindeguy C. Kurouski D. (2020b). Non-invasive diagnostics of Liberibacter disease on tomatoes using a hand-held Raman spectrometer. Planta 251:64. doi: 10.1007/s00425-020-03359-5, PMID: 32048047

[ref31] Sanchez L. Pant S. Irey M. S. Mandadi K. Kurouski D. (2019a). Detection and identification of canker and blight on Orange trees using a hand-held Raman spectrometer. J. Raman Spectrosc. 50, 1875–1880. doi: 10.1002/jrs.5741

[ref32] Sanchez L. Pant S. Mandadi K. Kurouski D. (2020c). Raman spectroscopy Vs quantitative polymerase chain reaction in early stage Huanglongbing diagnostics. Sci. Rep. 10:10101. doi: 10.1038/s41598-020-67148-6, PMID: 32572139 PMC7308309

[ref33] Sanchez L. Pant S. Xing Z. Mandadi K. Kurouski D. (2019b). Rapid and noninvasive diagnostics of Huanglongbing and nutrient deficits on Citrus trees with a handheld Raman spectrometer. Anal. Bioanal. Chem. 411, 3125–3133. doi: 10.1007/s00216-00019-01776-00214, PMID: 30989272

[ref34] Schaad N. W. Frederick R. D. Shaw J. Schneider W. L. Hickson R. Petrillo M. D.. (2003). Advances in molecular-based diagnostics in meeting crop biosecurity and Phytosanitary issues. Annu. Rev. Phytopathol. 41, 305–324. doi: 10.1146/annurev.phyto.41.052002.095435 14527331

[ref35] Stover R. H. (1962). Fusarial Wilt (Panama Disease) of Bananas and Other Musa Species. Kew, United Kingdom: Commonwealth Mycological Insititute.

[ref36] Synytsya A. Čopíková J. Matějka P. Machovič V. (2003). Fourier transform Raman and infrared spectroscopy of Pectins. Carbohydr. Polym. 54, 97–106. doi: 10.1016/S0144-8617(03)00158-9

[ref37] Tschirner N. Brose K. Schenderlein M. Zouni A. Schlodder E. Mroginski M. A.. (2009). The anomaly of the ν1-resonance Raman band of β-carotene in solution and in photosystem I and II. Physica Status Solidi 246, 2790–2793. doi: 10.1002/pssb.200982299

[ref38] Virkler K. Lednev I. K. (2009). Analysis of body fluids for forensic purposes: from laboratory testing to non-destructive rapid confirmatory identification at a crime scene. Forensic Sci. Int. 188, 1–17. doi: 10.1016/j.forsciint.2009.02.013, PMID: 19328638

[ref39] Yeturu S. Vargas Jentzsch P. Ciobotă V. Guerrero R. Garrido P. Ramos L. A. (2016). Handheld Raman spectroscopy for the early detection of plant diseases: Abutilon mosaic virus infecting Abutilon Sp. Anal. Methods 8, 3450–3457. doi: 10.1039/c6ay00381h

[ref40] Yu M. M. Schulze H. G. Jetter R. Blades M. W. Turner R. F. (2007). Raman microspectroscopic analysis of Triterpenoids found in plant cuticles. Appl. Spectrosc. 61, 32–37. doi: 10.1366/000370207779701352, PMID: 17311714

